# Comparison of the pharmacokinetics, safety and tolerability of two concentrations of a new liquid recombinant human growth hormone formulation versus the freeze-dried formulation

**DOI:** 10.1186/1472-6904-10-14

**Published:** 2010-10-20

**Authors:** Bernd Liedert, Ulf Forssmann, Peter Wolna, Michaela Golob, Andreas Kovar

**Affiliations:** 1 Merck KGaA, Darmstadt, Germany; 2 Merck Serono S.A. - Geneva, Switzerland (an affiliate of Merck KGaA, Darmstadt, Germany; 3 Merck KGaA, Grafing, Germany

## Abstract

**Background:**

Somatropin is recombinant human growth hormone (GH) used for the treatment of growth failure in children and GH deficiency in adults. Two concentrations of a liquid formulation have been developed: 5.83 and 8.0 mg/mL. This trial compared the pharmacokinetics (PK), safety and tolerability of these two liquid concentrations against the freeze-dried (FD) formulation in healthy volunteers.

**Methods:**

In an open-label, single-centre, three-way crossover study, volunteers (aged 18-45 years) were given subcutaneous injections of the reconstituted FD and two liquid formulations in random sequential order, each at 4 mg/dose, with a 1-week wash-out period between doses. To suppress endogenous GH secretion, intravenous somatostatin was infused continuously 1 hour before to 24 hours after each dose, achieving a cumulative dose of 3 mg. Primary PK endpoints were area under the serum concentration-time curve (AUC_0-t_) and maximum serum concentration (C_max_). For each of the two liquid formulations, bioequivalence with the FD formulation was concluded if the 95% confidence intervals (CIs) for the estimated test/reference ratios of geometric means of AUC_0-t _and C_max _were within the standard pre-specified acceptance range (0.80-1.25).

**Results:**

Fifteen men and 15 women enrolled (safety population, n = 30; PK population, n = 28). Bioequivalence with the FD formulation could be shown for both liquid formulations. The ratios of geometric means (95% CI) were 1.046 (0.980, 1.117) and 0.991 (0.929, 1.058) for AUC_0-t _and 0.954 (0.875, 1.040) and 0.955 (0.876, 1.041) for C_max _for the 5.83 and 8.0 mg/mL formulations, respectively. No significant differences between the three treatments in half-lives, time to reach C_max_, clearance or volume of distribution were observed. After injection, the most common side-effects were pain or injection-site reactions (all of mild intensity). There were no clinically significant abnormal vital signs, ECG or laboratory findings. There were 56 treatment-related adverse events (AEs): 49 mild, 6 moderate and 1 severe (vomiting). No serious AEs occurred. The pattern of AEs was as expected and all resolved by study end.

**Conclusion:**

Both concentrations of a new liquid multi-dose formulation are bioequivalent to the FD reference formulation and all are well tolerated.

**Trial registration number:**

NCT01034735.

## Background

Recombinant human growth hormone (r-hGH) is used for the treatment of children with growth failure due to inadequate secretion of endogenous GH, gonadal dysgenesis (Turner syndrome) or chronic kidney disease, and for short children born small for gestational age. It is also indicated for the treatment of GH deficiency (GHD) in adults. Long-term treatment is effective at promoting growth in GH-deficient children [1-7]. In a retrospective survey of 631 children, r-hGH increased height by a mean of approximately 8 cm per year over 2 years [3]. Similarly, in an open-label study of 69 children with organic or idiopathic GHD, r-hGH was associated with a median growth of 47.5 ± 8.5 cm over 7 years of treatment, with most subjects reaching their predicted final height [1].

Saizen^® ^(Merck Serono S.A. - Geneva, Switzerland, an affiliate of Merck KGaA, Darmstadt, Germany) r-hGH is available as a freeze-dried (FD) multi-dose formulation that needs to be reconstituted with bacteriostatic water before use. Once reconstituted, it is administered from a multi-dose cartridge, either by needle and syringe or by needle-free jet injection. To eliminate this need for reconstitution, a ready-to-use liquid multi-dose formulation is in development, which will avoid any risk of contamination or dilution errors during preparation and increase the convenience of drug administration. Furthermore, increasing the ease of injecting r-hGH may help to improve adherence rates (although this has yet to be demonstrated directly). Non-adherence is a problem with all long-term treatments, and for children taking r-hGH manifests as a lower growth rate in poorly compliant children compared with children who miss fewer injections [3,8].

The new liquid formulation is being developed in two concentrations: 5.83 and 8.0 mg/mL. This trial is the first comparison of the pharmacokinetics (PK), safety and tolerability of the two concentrations against the FD formulation in healthy volunteers.

## Methods

### Participants

The study enrolled healthy male and female volunteers. Eligible subjects were required to be aged 18-45 years; have a body weight greater than 55 kg and a body mass index (BMI) of > 20 and ≤ 30 kg/m^2^; have vital signs within the normal range; and to be either non-smokers or smoke fewer than 10 cigarettes per day. Females were also required to have a negative serum pregnancy test within 3 weeks of the trial start and a negative urine pregnancy test at the day before dosing. The following exclusion criteria were applied: a history or presence of cholelithiasis, diabetes, tumours in the pituitary gland or hypothalamus, any serious allergy, positive serological test for hepatitis B or C and HIV, hypertension or other significant cardiovascular abnormality. Subjects were also excluded if they had a significant history or clinical evidence of auto-immune, gastrointestinal, haematological, haematopoietic, hepatic, neurological, pancreatic or renal disease, or had a positive drug or alcohol test or chronic use of medication.

### Study design

The single-centre trial (NCT01034735), carried out at a clinical pharmacology research centre in Germany, had an open, randomized, three-way crossover design. Treatment with r-hGH (Saizen^®^; Merck Serono S.A. - Geneva) started within 21 days of screening. Each volunteer received three treatments: reconstituted FD (8.8 mg/1.51 mL) and two liquid formulations (5.83 and 8.0 mg/mL). All three treatments were administered as a single subcutaneous dose of 4 mg, to allow proper determination of the PK parameters. The doses were administered in a randomized sequence with a 1-week wash-out period between each administration. The doses were injected into the anterior abdominal wall, around the umbilicus at the top of a skin fold, using a needle and syringe, and were injected at a distance of at least 10 cm from one another in pre-marked locations.

Additionally, intravenous somatostatin was infused continuously from 1 hour before to 24 hours after each dose, achieving a cumulative dose of 3 mg. This pituitary down-regulation was necessary to suppress endogenous GH secretion in the healthy volunteers to allow reliable calculation of PK parameters.

For each r-hGH administration, the subject attended the clinical unit on the day before the study drug administration (days -1, 7 and 14) and stayed in the unit until 26 hours after drug administration (days 2, 9 and 16). Blood samples for PK analysis were taken hourly up to 10 hours, and then at 12, 18 and 24 hours post-dose. Vital signs, safety and tolerability were assessed before and 24-26 hours after each dose and 14 ± 3 days after the last dose.

The trial was conducted in compliance with the Declaration of Helsinki and the ICH Guideline of Good Clinical Practice, in addition to European, US and German directives for proper conduct of clinical drug trials. The study was approved by the national regulatory agency (the Federal Institute for Drugs and Medicinal Products [Bundesinstitut für Arzneimittel und Medizinprodukte]) and the independent ethics committee of the Bavarian Chamber of Physicians. Participants gave written informed consent at the start of the trial.

### Objectives

The primary objective of the study was to assess the bioequivalence of two concentrations of the liquid formulation of r-hGH in comparison with the FD formulation. The secondary objectives were to evaluate safety and tolerability, and to describe the PK parameters of the liquid formulations of r-hGH in comparison with the FD formulation.

### Outcomes measures

Primary PK endpoints were the area under the serum concentration-time curve from time 0 to the last measurable concentration time point after drug administration (AUC_0-t_) and the maximum serum concentration (C_max_). Secondary PK endpoints included the total area under the serum concentration-time curve extrapolated to infinity (AUC_0-∞_), time to reach C_max _(t_max_), terminal elimination half life (t_1/2_), apparent volume of distribution (V_z/f_) and apparent clearance (CL/f).

Quantitative determination of r-hGH in human serum samples was performed using a 'two-step' sandwich-type immunoassay. The assay was fully validated according to the FDA and EMA guidelines on bioanalytical method validation and best practice recommendations for the validation and implementation of quantitative bioanalytical methods [9-11]. The lower limit of quantification (LLOQ) of the immunoassay was 0.4 ng/mL. The actual administered dose was determined by gravimetric measurement, subtracting the post-dose weight of the syringe from the pre-dose weight and calculating with a density of 1.02 g/mL for the 5.83 mg/mL liquid formulation and FD formulation and 1.03 g/mL for the 8 mg/mL liquid formulation.

Tolerability was assessed by adverse events (AEs) queried on an ongoing basis from the day prior to dosing to at least 14 ± 3 days after the last dose administration. Additionally, tolerability was evaluated immediately pre-dose, at the time of administration (injection-site reactions [ISRs] only), 5 minutes, and 2, 4, 6, 12 and 24 hours after each injection. ISRs were assessed by the investigator, whereas itching at the injection site and pain were assessed by the subject; a 100 mm visual analogue scale (VAS) was used to assess pain.

Safety assessments comprised physical examination, vital signs (blood pressure, pulse rate, body temperature), supine 12-lead ECG recordings and laboratory tests. The physical examination was made at screening and at 14 ± 3 days after each administration. Vital signs were taken immediately pre-dose, 1, 6, 12 and 24 hours after treatment administration, and at the post-study visit. ECG measurements were taken before each dose.

### Sample size

A total of 24 evaluable subjects (four subjects for each treatment sequence) were required to provide approximately 90% power for demonstrating bioequivalence. This calculation assumed intra-subject coefficients of variation of approximately 12% for AUC and 17% for C_max_, based on earlier studies with the FD formulation and values between 0.95 and 1.05 for the true treatment ratio test/reference. The 95% confidence level (CI) was used to adjust for multiplicity. To account for potential drop-outs, 30 subjects were enrolled.

### Randomization

Subjects were randomly assigned in chronological order to a treatment sequence, according to the randomization list, with an equal number of subjects in each of the six treatment sequences. Randomization of each subject to a treatment sequence occurred immediately before dosing on study day 1.

### PK analysis and statistical methods

PK analyses were performed for the PK population and the safety analyses were performed for the safety population. The PK population included all subjects who had been treated according to the protocol in all trial periods who fulfilled the following criteria: all inclusion/exclusion criteria satisfied; adequate trial medication compliance; absence of relevant protocol violations and availability for the primary target variables for at least one treatment. The safety population included all subjects who received at least one dose of trial medication and had follow-up safety data.

The PK parameters of r-hGH were calculated according to non-compartmental standard methods using the validated computer program KINETICA (Version 4.4.1, Thermo, Philadelphia, USA). Statistical analysis was performed using the computer program package SAS System for Windows (TM version 8.2; SAS Institute, Cary NC, USA).

A mixed model was fitted to each of the log-transformed PK endpoints, C_max_, AUC_0-t_, C_max _adjusted to target dose (C_max adj_) and AUC_0-t _adjusted to target dose (AUC_0-t adj_) with fixed effect terms for treatment, period and sequence, and subject as a random effect. In compliance with the relevant FDA and EMA guidelines [9-11], for each of the two test liquid formulations, bioequivalence was concluded if the 95% CIs for the estimated test/reference ratios of geometric means of AUC_0-t _and C_max _were within the standard pre-specified acceptance range for bioequivalence (0.80-1.25). In addition, a further correction for the measured active content (as determined by the certificate of analysis of each formulation) was applied to the ratio estimates and confidence limits in order to further assess bioequivalence (Table 1).

**Table 1:** Actual dose and summary of calculation of active content as a percentage of target (n = 28, pharmacokinetic population)

Parameter	Formulation		
	
	A	B	C
Actual dose (geometric mean [CV%] mg)	3.695 (3.26)	3.591 (2.92)	3.662 (3.95)
Active content (AC), mg	6.0	7.7	8.2
Container volume (V), mL	1.03	1.0	-
Concentration (AC/V), mg/mL	5.825	7.7	5.43
Target concentration (T), mg/mL	5.83	8.0	-
Percentage of target concentration A and B: 100 × [AC/V]/T C: 100 × [AC/T]	99.92	96.25	93.18

CV, coefficient of variation; r-hGH, recombinant human growth hormone.

A = r-hGH liquid multi-dose, 5.83 mg/mL; injection of 0.686 mL.

B = r-hGH liquid multi-dose, 8.0 mg/mL; injection of 0.5 mL.

C = r-hGH freeze-dried 8.8 mg, reconstituted in 1.51 mL; injection of 0.686 mL.

All PK parameters were presented in a descriptive way per treatment group (number of subjects [n], mean, standard deviation [SD], median, geometric mean, standard error of mean [SEM], coefficient of variation, minimum [Min] and maximum [Max] values). Mean serum concentrations were also described per time point and treatment. Values below the LLOQ were taken as zero for descriptive statistics of concentrations.

The numerical values for tolerability and safety variables were summarized descriptively for the safety population.

## Results

### Participants

Fifteen men and 15 women were enrolled. Two subjects dropped out at the time of check-in for the third period due to non-adherence to protocol requirements. The mean age of the volunteers was 31.8 years (range 18-45 years). At the pre-study examination, the mean weight was 70.4 kg (54.9-91.8 kg), BMI was 23.7 (20.1-28.7) and height was 1.72 m (1.54-1.89 m). The characteristics of subjects were similar across the treatment sequences: the mean age range was 24.2-38.0 years, mean weight 65.0-76.5 kg and mean BMI 23.0-24.8 kg/m^2^, and the number of subjects per sequence ranged between 1 and 4. In total, 21 volunteers were non-smokers and nine were smokers.

The PK population comprised 28 subjects, and all 30 subjects received at least one dose of trial medication, so were included in the safety evaluation.

### Pharmacokinetics

Bioequivalence to the FD formulation could be demonstrated for both concentrations of the liquid-formulation treatment as the 95% CI for the ratios of geometric means for AUC_0-t _and C_max _fell within the acceptance criteria of 0.80-1.25. The actual dose of the treatments administered varied slightly between the FD formulation and the two concentrations of the liquid formulation (Table 1). However, adjustments for actual dose administered, active content, or both, further supported the conclusion of bioequivalence (Table 2).

**Table 2:** Estimates with 95% confidence intervals for ratios of geometric means in pharmacokinetic (PK) parameters of recombinant human growth hormone (r-hGH) in serum (n = 28, PK population)

Statistic	Formulation	AUC_0-t_, ng/mL*h	C_max_, ng/mL	AUC_0-t adj_, ng/mL*h	C_max adj_, ng/mL
				
				Adjusted for actual dose	
Least squares geometric mean	A	293.1	39.52	317.4	42.80
	B	277.7	39.57	309.5	44.09
	C	280.1	41.43	306.1	45.26
CV%		12.2	16.2	12.3	16.2
Ratio estimate (95% CI)	A/C	1.05 (0.98, 1.12)	0.95 (0.88, 1.04)	1.04 (0.97, 1.11)	0.95 (0.87, 1.03)
	B/C	0.99 (0.93, 1.06)	0.96 (0.88, 1.04)	1.01 (0.95, 1.08)	0.97 (0.89, 1.06)
Ratio estimate (95% CI) (corrected for active content)	A/C^a^	0.98 (0.91, 1.04)	0.89 (0.82, 0.97)	0.97 (0.91, 1.03)	0.88 (0.81, 0.96)
	B/C^b^	0.96 (0.90, 1.02)	0.93 (0.85, 1.01)	0.98 (0.92, 1.05)	0.94 (0.87, 1.03)

^a^Correction factor: 93.18/99.9; ^b^Correction factor: 93.18/96.25.

A = r-hGH liquid multi-dose, 5.83 mg/mL.

B = r-hGH liquid multi-dose, 8.0 mg/mL.

C = r-hGH freeze-dried 8.8 mg, reconstituted in 1.51 mL.

AUC_0-t_, area under the serum concentration-time curve from time 0 to the last measurable concentration time point after drug administration; C_max_, maximum serum concentration; CI, confidence interval;

CV, coefficient of variation.

**Figure 1:**
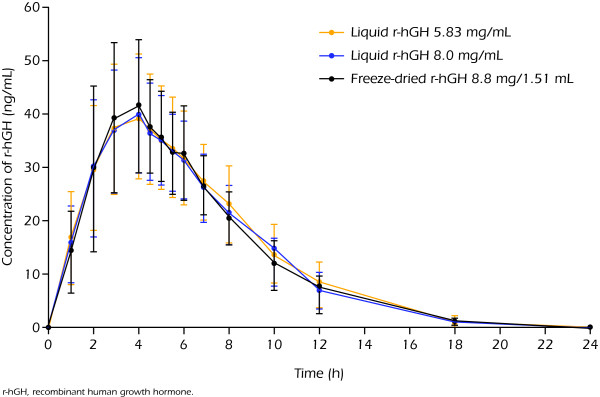
**Mean concentration-time profile of r-hGH in serum (n = 28). Administered dose of 4 mg**.

All pre-dose serum samples had concentrations of GH below the LLOQ. Overall, the PK parameters of the two strengths of liquid formulation and the FD formulation of r-hGH showed low variability. There were no significant differences between the three treatments in half-lives (t_1/2_), time to reach C_max _(t_max_), clearance (CL/f) or volume of distribution (V_z/f_) (Table 3). The least squares geometric means in Table 2 are derived from the mixed model. They deviate very slightly from the ordinary geometric means in Table 3, therefore. The mean concentration-time profiles showed low and similar intra-subject variability and were close to superimposable for the three treatments (Figure 1).

**Table 3:** Pharmacokinetic (PK) parameters of recombinant human growth hormone (r-hGH) in serum (n = 28, PK population)

Parameter	Formulation		
	
	A	B	C
AUC_0-t_, ng/mL*h	295 (23.1) 191-474	279 (26.2) 120-381	281 (23.6) 165-411
C_max_, ng/mL	39.8 (28.0) 21.9-70.9	39.8 (31.6) 18.9-67.8	41.6 (31.1) 18.4-75.6
AUC_0-∞_, ng/mL*h	306 (23.3) 200-480	291 (24.2) 135-386	291 (23.3) 170-418
t_max_, h	4.0 2.0-7.0	4.0 2.0-6.0	4.0 2.0-6.0
t_1/2_, h	2.19 (32.0) 1.24-5.38	2.22 (27.2) 1.42-4.11	2.05 (30.2) 1.18-3.85
V_z/f_, L	41.2 (41.3) 18.3-117.9	44.0 (45.3) 24.2-132.5	40.7 (40.5) 19.8-90.6
CL/f, L/h	13.1 (23.3) 8.3-20.0	13.7 (24.2) 10.4-29.5	13.8 (23.3) 9.6-23.5

Geomean (geo CV%) and range, for t_max _median and range are given.

AUC_0-t_, area under the serum concentration-time curve from time 0 to the last measurable concentration time point after drug administration; AUC_0-∞_, total area under the serum concentration-time curve extrapolated to infinity; C_max_, maximum serum concentration; CL/f, apparent clearance; CV, coefficient of variation; t_1/2_, terminal elimination half life; t_max_, time to reach C_max_; V_z/f_, apparent volume of distribution.

A = r-hGH liquid multi-dose, 5.83 mg/mL.

B = r-hGH liquid multi-dose, 8.0 mg/mL.

C = r-hGH freeze-dried 8.8 mg, reconstituted in 1.51 mL.

### Tolerability and safety

Twenty-six subjects (87%) reported pain after drug administration. Most of the episodes were very mild, with a mean intensity of only 3.52 mm on the 100 mm VAS and a maximum of 29 mm. The pain typically resolved within 5 minutes of injection.

A total of 31 ISRs occurred in 19 subjects (Table 4). Reactions comprised redness, bruising and itching. Fourteen, eight and nine ISRs occurred after treatment with the 5.83 mg/mL liquid, 8.0 mg/mL liquid and FD formulations, respectively. Twenty-six of the reactions were recorded 5 minutes post-dose. All reactions were mild, with no local reactions considered moderate or severe.

**Table 4:** Injection-site reactions, by number of events (n = 30, safety population)

	Formulation			Overall
		
	A	B	C	
Redness	12	7	8	27
Bruising	1	0	1	2
Itching	1	1	0	2
Swelling	0	0	0	0
Induration	0	0	0	0
Total	14	8	9	31

r-hGH, recombinant human growth hormone.

A = r-hGH liquid multi-dose, 5.83 mg/mL.

B = r-hGH liquid multi-dose, 8.0 mg/mL.

C = r-hGH freeze-dried 8.8 mg, reconstituted in 1.51 mL.

AEs were experienced by 26/30 (87%) subjects. There were 95 AEs overall (four pre-dose) with 56 considered as treatment-related (Table 5). Of the treatment-related AEs, 49 were mild in intensity, with only six instances of moderate AEs and one case of severe vomiting. There were no serious or life-threatening AEs and all events resolved by the end of the study.

**Table 5:** Treatment-emergent and treatment-related adverse events (AEs) (n = 30, safety population)

	Formulation			Overall
		
	A	B	C	
Subjects experiencing a treatment-emergent AE, n (%)	19/30 (63.3)	16/29 (55.2)	18/29 (62.1)	26/30 (86.7)
Treatment-emergent AEs, n	35	27	29	91
*Treatment-related AEs*				
Total, n	24	12	20	56
Nausea	12	4	9	25
Headache	2	4	5	11
Dizziness	4	2	2	8
Vomiting	2	0	1	3
Upper abdominal pain	0	1	0	1
Abdominal pain	1	0	0	1
Peripheral oedema	1	0	0	1
Abdominal discomfort	0	0	1	1
Tachycardia	1	0	0	1
Pain in extremity	1	0	0	1
Asthenia	0	1	0	1
Injection site bruising	0	0	1	1
Tremor	0	0	1	1

r-hGH, recombinant human growth hormone.

A = r-hGH liquid multi-dose, 5.83 mg/mL.

B = r-hGH liquid multi-dose, 8.0 mg/mL.

C = r-hGH freeze-dried 8.8 mg, reconstituted in 1.51 mL.

There were no clinically significant abnormal vital signs or ECG results. All laboratory values were within the reference range or were judged by the investigator not to be clinically relevant.

## Discussion

This study has shown that both concentrations of a new liquid formulation were bioequivalent to the FD reference formulation in healthy volunteers with pituitary somatrope cell down-regulation. This conclusion is robust to adjustments to each subject's actual dose and to adjustments for active content. There were no apparent differences between the two liquid formulations of r-hGH and the FD formulation in the rate and extent of drug exposure, half-life, clearance or volume of distribution. In addition, variability in the PK parameters was low across the three treatments.

Demonstration of bioequivalence of different formulations is not assumed to be significantly influenced by characteristics of the study population. Because of this, data from healthy volunteers can be extrapolated to adult as well as child patients.

The r-hGH liquid multi-dose and the r-hGH FD formulations were administered at a dose of 4 mg. This dose level is far higher than the recommended therapeutic dose in adults (which is started at 0.15-0.3 mg/day and seldom exceeds 1.0 mg/day) or children (which does not exceed 0.05 mg/kg body weight/day) [12], but was chosen to allow accurate determination of GH PK parameters. Pituitary down-regulation was also necessary to suppress endogenous GH secretion in the healthy volunteers and to reliably calculate the PK parameters. The dose of 3 mg somatostatin in 25 hours was based on previous experience [13,14] and was also higher than therapeutic doses (which do not usually exceed 0.6 mg/day for adults [15,16]).

The high doses of drugs given led to higher rates of AEs than expected in clinical practice. Most of the AEs that were reported, including nausea, vomiting, headache and dizziness, can be related to the somatostatin infusion [1,2,12-16]. No serious AEs were reported. Both test preparations were well tolerated with regard to local site reactions, vital signs, ECG findings and laboratory findings, as was the reference FD preparation. Post-injection pain was mild and transient and any ISRs were similarly low in intensity.

The label for the liquid r-hGH is expected to be the same as for the FD r-hGH with the exception of the storage requirements. Although both the liquid and FD formulations (before reconstitution) have a shelf life of 2 years, the liquid must be stored at 2-8°C, whereas the FD formulation can be stored at < 25°C. The shelf-life and storage conditions after the cartridges have started to be used are the same across formulations: 21-28 days at 2-8°C (depending on the local label). Both liquid formulations will be available in 3 mL cartridges with stability for at least 18 months at 2-8°C, allowing inter-changeability between the electronic and needle-free delivery devices. It is hoped that the simplification of the injection or needle-free administration process provided by liquid r-hGH will make this treatment less of a burden for patients. Increasing the ease of injecting r-hGH may help to make treatment more convenient for patients, thereby improving adherence rates.

## Conclusion

The two strengths of the liquid multi-dose formulation of r-hGH are bioequivalent to the FD multi-dose reference formulation and are well tolerated. This conclusion is robust to adjustments for the active content and the actual dose administered.

## Competing interests

All authors are employees of Merck KGaA, Darmstadt or Grafing, Germany.

## Authors' contributions

BL was study director in the terminal phase of the study, and was involved in the data analysis, interpretation and reporting of results. UF was involved in the design and conduct of the study, performed the medical supervision and contributed to the data analysis, interpretation and reporting of results. PW drafted the statistical design of the study, performed power calculations and was involved in the data analysis and interpretation of results. MG carried out bioanalytical assay development and validation followed by sample analysis and provided bioanalytical data for evaluation. AK was involved in and responsible for the study design and PK data analysis, and contributed to the interpretation and reporting of results. All authors read, reviewed and approved the final manuscript.

## Pre-publication history

The pre-publication history for this paper can be accessed here:

http://www.biomedcentral.com/1472-6904/10/14/prepub
